# Sensory nerve‐deficient microenvironment impairs tooth homeostasis by inducing apoptosis of dental pulp stem cells

**DOI:** 10.1111/cpr.12803

**Published:** 2020-04-04

**Authors:** An‐Qi Liu, Li‐Shu Zhang, Dong‐Dong Fei, Hao Guo, Mei‐Ling Wu, Jin Liu, Xiao‐Ning He, Yong‐Jie Zhang, Kun Xuan, Bei Li

**Affiliations:** ^1^ State Key Laboratory of Military Stomatology & National Clinical Research Center for Oral Diseases & Shaanxi International Joint Research Center for Oral Diseases Center for Tissue Engineering School of Stomatology The Fourth Military Medical University Xi'an China; ^2^ Xi'an Institute of Tissue Engineering and Regenerative Medicine Xi'an China; ^3^ Department of Orthodontic Dentistry School of Stomatology The Fourth Military Medical University Xi'an China; ^4^ Department of Periodontic Dentistry School of Stomatology The Fourth Military Medical University Xi'an China; ^5^ Department of Preventive Dentistry School of Stomatology The Fourth Military Medical University Xi'an China

**Keywords:** dental pulp stem cells, inferior alveolar nerve, microenvironment, nervous system, tooth

## Abstract

**Objectives:**

The aim of this study is to investigate the role of sensory nerve in tooth homeostasis and its effect on mesenchymal stromal/stem cells (MSCs) in dental pulp.

**Materials and methods:**

We established the rat denervated incisor models to identify the morphological and histological changes of tooth. The groups were as follows: IANx (inferior alveolar nerve section), SCGx (superior cervical ganglion removal), IANx + SCGx and Sham group. The biological behaviour of dental pulp stromal/stem cells (DPSCs) was evaluated. Finally, we applied activin B to DPSCs from sensory nerve‐deficient microenvironment to analyse the changes of proliferation and apoptosis.

**Results:**

Incisor of IANx and IANx + SCGx groups exhibited obvious disorganized tooth structure, while SCGx group only showed slight decrease of dentin thickness, implying sensory nerve, not sympathetic nerve, contributes to the tooth homeostasis. Moreover, we found sensory nerve injury led to disfunction of DPSCs via activin B/SMAD2/3 signalling in vitro. Supplementing activin B promoted proliferation and reduced apoptosis of DPSCs in sensory nerve‐deficient microenvironment.

**Conclusions:**

This research first demonstrates that sensory nerve‐deficient microenvironment impairs tooth haemostasis by inducing apoptosis of DPSCs via activin B/SMAD2/3 signalling. Our study provides the evidence for the crucial role of sensory nerve in tooth homeostasis.

## 1 INTRODUCTION

Nerve putatively plays a critical role in tissue homeostasis and regeneration, such as bone homeostasis maintaining^1^ and salamander limb regeneration.^2^ However, how nerve modulates tooth homeostasis remains largely unknown. Our team previously has found abnormal morphology of teeth in a patient with congenital insensitivity to pain with anhidrosis (CIPA), which is a rare inherited disorder of the peripheral nervous system. The patient displays severe oral manifestations, such as dentin hypoplasia and cementogenesis defects, implying the indispensable role of sensory nerve in tooth development.^3^ The rodent incisor is mainly innervated by sensory nerve and sympathetic nerve, which is derived from mandibular inferior alveolar nerve (IAN)^4^ and sympathetic superior cervical ganglion (SCG) respectively.^5^ It has been reported that impeding sensory nerve innervation leads to morphological aberrancy in mouse lower incisor,^6^ while which type of nerve mainly regulates incisor homeostasis has not been fully understood yet.

Nerve provides microenvironment for stromal/stem cells through complex signalling to dynamic regulate their behaviour, building highly elaborate structures and thus maintaining tissue homeostasis.^7^, ^8^ Studies have illustrated that denervation impedes cellular behaviour in various tissues,^8^, ^9^ but whether and how denervation impairs odontogenic stromal/stem cells responsible for tooth homeostasis remains elusive. The rodent lower incisor as an independent organ grow continuously, offering an excellent model to address the relationship between nerve and odontogenic stromal/stem cells.^10^ Additionally, dental pulp stromal/stem cells (DPSCs) as a kind of mesenchymal stromal/stem cells (MSCs) in tooth can differentiate into odontoblasts and pulp cells participating in tooth homeostasis and regeneration.^11^, ^12^, ^13^ After sensing injury stimuli, the stem cells in pulp proliferate and migrate to the injured area to maintain tooth homeostasis.^11^ In the field of tissue engineering, DPSCs exhibit excellent capacities of proliferation and differentiation when combined with scaffolds such as nanoporous, hydrogel and mesoporous silicon scaffolds; thus, they have been widespread applied in tooth, bone and nerve regeneration procedures.^14^ For tissue regeneration, many innovative scaffolds have been proved beneficial to stem cell‐based tissue regeneration, including nanosilicates which are mineral‐based two‐dimensional nanomaterials, graphene which is utilized to create three‐dimensional porous foams and bioactive nanomaterial delivery system which can release bioactive molecule as needed.^15^, ^16^ However, the direct effect of nerve‐deficient microenvironment on DPSCs is largely unclear. Understanding this will shed light on the regeneration mechanism of incisor and further provide novel perspective of pulp regeneration.

In this study, we used rat denervated models to demonstrate that sensory nerve, not sympathetic nerve, maintains tooth homeostasis and dentin formation. Moreover, through isolating rat DPSCs from sensory denervated incisor (IANx‐DPSCs), we found denervation led to DPSCs disfunction. Sensory nerve injury induced apoptosis of DPSCs via activin B/SMAD2/3 signalling. Supplementing activin B promoted proliferation and reduced apoptosis of DPSCs in sensory nerve‐deficient microenvironment.

## 2 MATERIALS AND METHODS

### 2.1 Animals

All animal experiments were performed following the guidelines of the Intramural Animal Use and Care Committee of the Fourth Military Medical University, Xi'an, China. All animals were purchased from Animal Center of Fourth Military Medical University, Xi'an, China. Four‐week‐old female Sprague Dawley (SD) rats were used for DPSCs isolation. Six‐week‐old female SD rats were used for histomorphology assay.

Six‐week‐old SD rats were distributed into four groups: IANx group (sectioning the unilateral IAN), SCGx group (removing the unilateral SCG), IANx + SCGx group (sectioning the unilateral IAN and removing the ipsilateral SCG) and the Sham group (performing the same surgical procedures except for resection of the nerve).

The denervation procedure had no impact on food or water uptake of the rat. Each group of rats was, respectively, sacrificed after 1, 2, 3 weeks through heart infusion, then collected the mandible and fixed them in 4% paraformaldehyde.

Four‐week‐old SD rats were distributed into Sham and IANx groups. Two weeks after surgery, the DPSCs were obtained from Sham and IANx group.

### 2.2 Animal surgery

#### 2.2.1 Inferior alveolar nerve axotomy (IANx)

**FIGURE A1:**
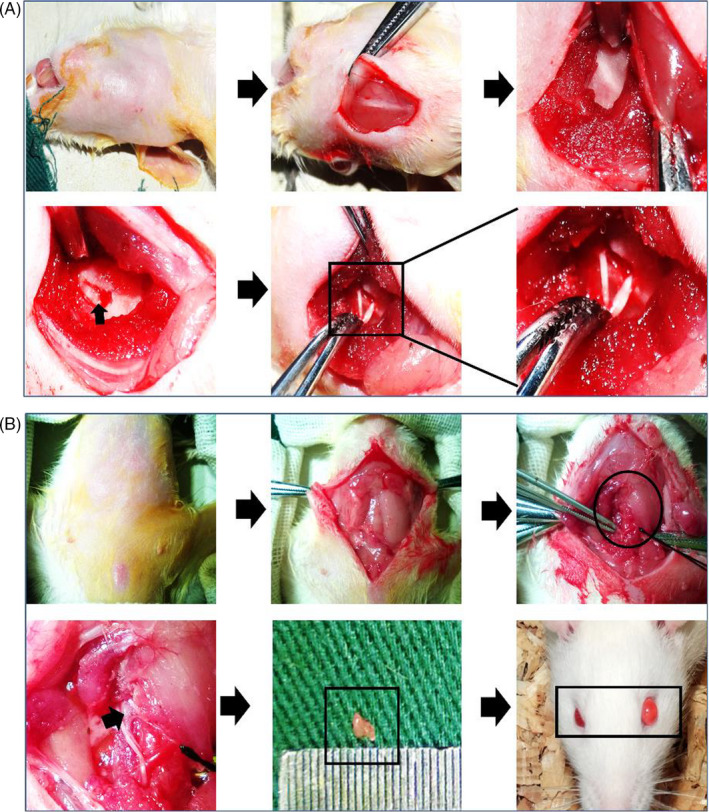
Animal surgery. A, The surgery process of Inferior alveolar nerve axotomy (IANx). B, The surgery process of superior cervical ganglionectomy (SCGx)

The IANx surgery was severed as previously described.^17^ Under general anaesthesia, an extraoral horizontal incision was made to fully expose the masseter muscle. The bone surface of the mandible was exposed by blunt dissection of the masseter muscle. A small dental round bur was used to remove the cortex bone and expose the IAN. Then 5 mm length of IAN was removed. The muscle and skin were closed and sutured (Figure A1(A)).

#### 2.2.2 Superior cervical ganglionectomy (SCGx)

The SCGx surgery was performed as previously described.^18^ Under general anaesthesia, the neck muscles were exposed through a 4‐cm vertical incision of neck region. Then, forceps were applied to dissect the deep cervical fascia and partially remove carotid sheath. After separating the common carotid artery, the SCG which was behind the carotid bifurcation was removed. Ipsilateral blepharoptosis has been used as indicator of the successful removal of SCG (Figure A1(B)).

### 2.3 Micro‐CT and histological analysis

The samples were collected and analysed by micro‐CT (Siemens Inveon, Germany). The longitudinal images of the mandibular incisor were acquired through three‐dimensional reconstruction, and the height of contour of mandibular first molar mesial surface was served as a fixed position to obtain cross‐sections. The thickness of dentin was analysed via ImageJ 1.47 software. After micro‐CT scanning, all mandibles were decalcified by 17% ethylene diamine tetraacetic acid (EDTA) (Pulpdent), embedded with paraffin and sliced in the sagittal plane for haematoxylin and eosin (H&E) (Leica) and Masson trichrome staining (Baso). The thickness of dentin and enamel at the apical were used for statistical analysis.

### 2.4 Immunofluorescence staining

The immunofluorescence staining was performed as previously described.^19^ The following primary antibodies were used in our study: DSPP Antibody (Santa Cruz Biotechnology, sc‐73632, 1:50), Anti‐CGRP antibody (Abcam, ab81887, 1:100) and Anti‐TH antibody (Abcam, ab6211, 1:200).

### 2.5 Isolation and culture of dental pulp stem cells (DPSCs)

The dental pulp was extracted from the lower incisor after removing apical buds. The DPSCs were obtained with tissue and enzymic digestion. In particular, the pulp was minced into 1 mm^3^ and digested in 3 mg/mL collagenase I (Sigma‐Aldrich Corp) at 37°C, 5% CO_2_ for 1.5 hours. Cells were then plated evenly on 6‐well plates with α‐minimum essential medium (α‐MEM; Gibco BRL) supplemented with 20% foetal bovine serum (FBS; Gibco BRL). Recombinant Mouse activin B Protein (R&D systems, 8260‐AB) was applied to culture medium in IANx group after cells adhesion at 10 ng/mL as recommended.^20^

### 2.6 Colony‐forming assay

To assess the ability to produce colony‐forming unit (CFU), single‐cell suspensions of DPSCs from Sham and IANx groups (1 × 10^3^ cells) were, respectively, seeded into 10‐cm‐diameter culture dishes. After 14 days of cultivation, cells were fixed with 4% paraformaldehyde for 30 minutes and then stained with 1% toluidine blue.

### 2.7 Cell Counting Kit‐8 (CCK‐8) assay

DPSCs from Sham, IANx and IANx + activin B groups were, respectively, cultured in 96‐well plates (2 × 10^3^ cells/well). CCK‐8 assay was carried out for 1, 3, 5, 7, 9 days according to the cell counting kit (7sea biotech, cell counting kit, China) protocol. After 24 hours, 20 μL CCK‐8 reagent was added into every well and incubates cells for 2 hours. The absorbance was measured at 450 nm wavelength with a microplate reader (Epoch; BioTek).

### 2.8 Cell apoptosis analysis

Apoptosis was measured by Annexin V‐FITC Apoptosis Detection Kit (Beyotime, China) according to manufacturer's instructions. The DPSCs of passages 3 to 6 from Sham, IANx and IANx + activin B group were incubated in 195 μL Annexin V‐FITC binding buffer and 5 μL Annexin V‐FITC for 10 minutes at room temperature. After washing, the cells were resuspended with 190 μL Annexin V‐FITC binding buffer and 10 μL propidium iodide. The cells were analysed with flow cytometry (Beckman Coulter).

### 2.9 Osteogenic/adipogenic differentiation of dental pulp stem cells (DPSCs)

Induction of osteoblasts and adipocytes was performed as previously described.^21^, ^22^ After 28 days of osteogenic induction, the cells were characterized by Alizarin red S staining, and total RNA was extracted and analysed for the presence of osteogenic genes (*Alp, Runx2* and *Opn*) by quantitative real‐time PCR (qRT‐PCR).

After 21 days of adipocyte induction, the cells were characterized by Oil Red O staining, and total RNA was extracted and analysed for the presence of adipogenic genes (*Lpl* and *Ppar‐γ*) by qRT‐PCR. For quantification analysis, Oil Red O staining was analysed with isopropanol (Sigma) and measured with a microplate reader (Epoch; BioTek) at 520 nm.

### 2.10 Quantitative Real‐Time PCR (qRT‐PCR)

See Appendix 1.

### 2.11 Western blot analysis

See Appendix 1.

### 2.12 Statistical analysis

All data were expressed as mean (±SD) from at least three independent experiments and analysed by two‐tailed unpaired Student's *t *test or one‐way ANOVA test using GraphPad Prism 5.0. Values of *P *< .05 were considered statistically significant.

## 3 RESULTS

### 3.1 Sensory nerve mainly maintains the tooth homeostasis

**FIGURE 1:**
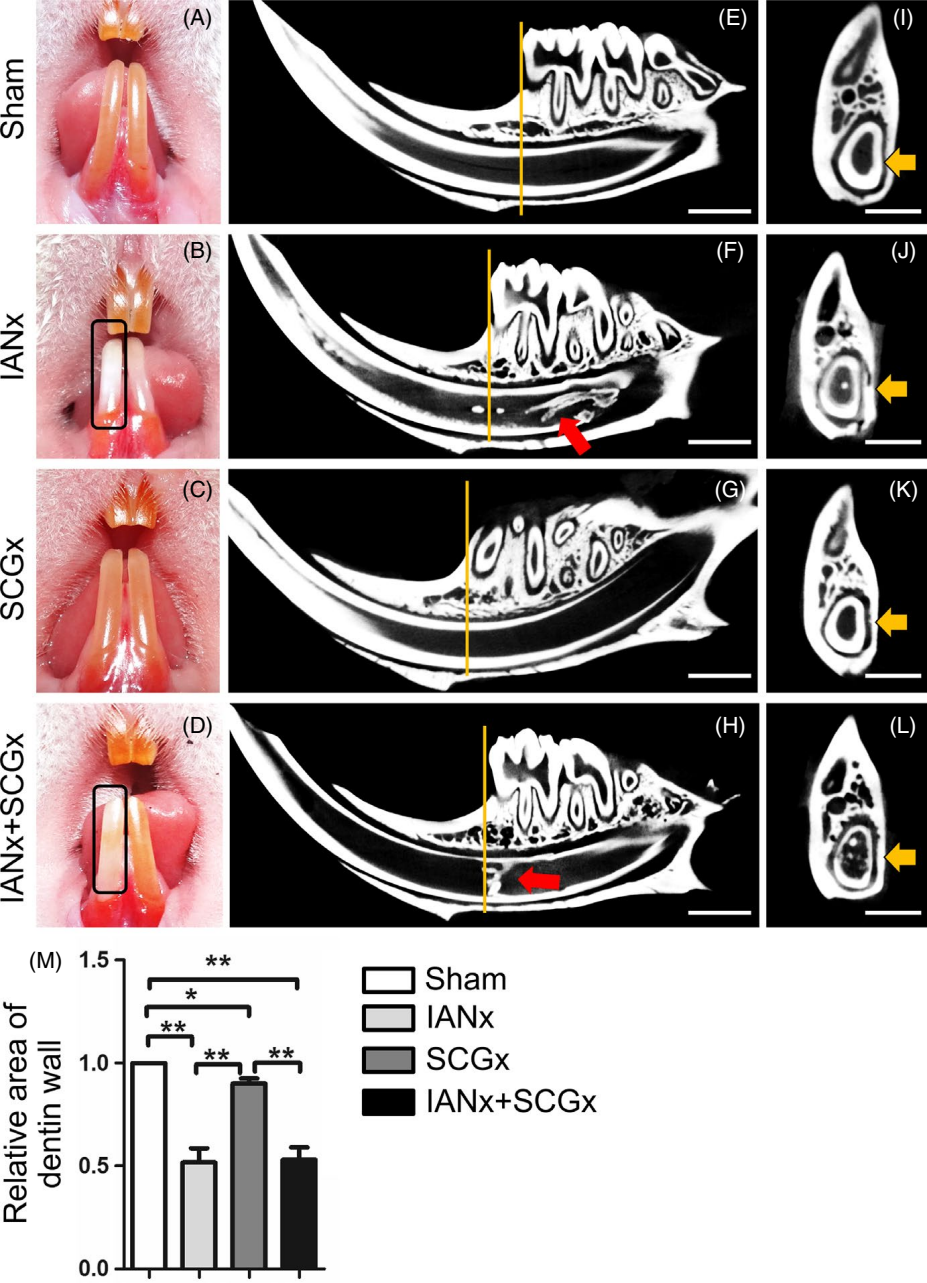
Sensory nerve maintains the phenotype of incisor. A‐D, The phenotype of rat incisors after 2 wk of surgery. The right incisor in IANx (B) and IANx + SCGx (D) groups turn chalky (indicated by boxes), while the Sham (A) and SCGx group (C) do not exhibit the similar phenotype. E‐H, The micro‐CT shows the longitudinal images of the incisors from four groups after 2 wk of surgery. The irregular calcified masses (indicated by red arrows) can be observed in IANx (F) and IANx + SCGx group (H), not in SCGx (G) or Sham group (E). Scale bar: 2 mm. I‐L, The cross‐sections are sampled at comparable positions, indicated by yellow lines in E‐H. Scale bar: 1 mm. The area of dentin walls (indicated by yellow arrows) is measured. M, Compared to Sham, the thickness of dentin in the other three groups displays a reduction. (*P < .05; **P < .01; n = 5). Besides, IANx and IANx + SCGx groups show thinner dentin wall than SCGx group. (**P < .01; n = 5)

**FIGURE 2:**
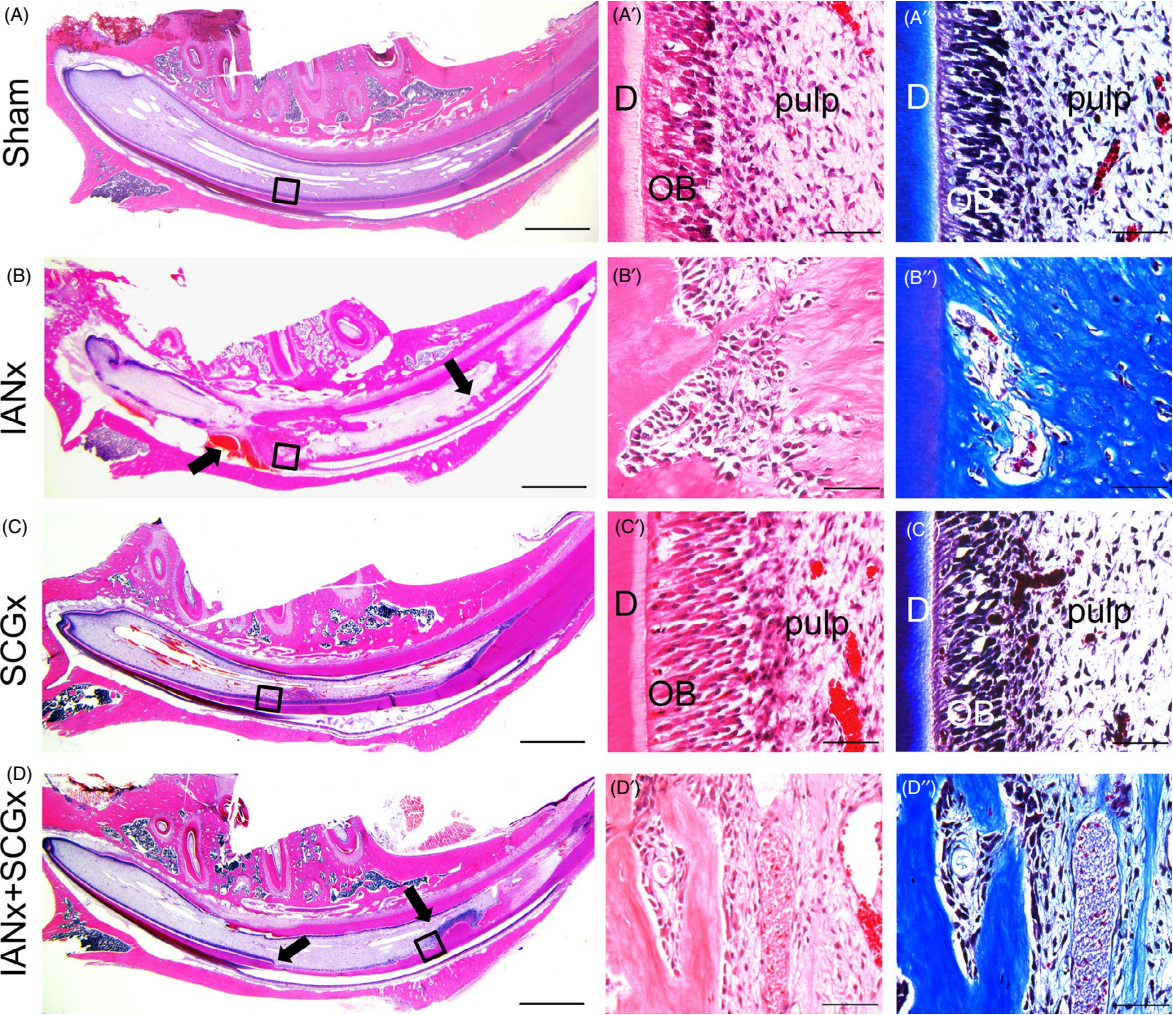
Sensory nerve maintains the tooth homeostasis. (A‐D) HE staining exhibits low magnification for each group. The IANx and IANx + SCGx incisors show disorganized structures (indicated by arrows). Scale bars: 2 mm. HE and Masson staining on the right represent the high magnification of boxed areas, displaying classical incisor structure in Sham (A′, A″) and SCGx (C′, C″) group, as well as disorganized structure in IANx (B′, B″) and IANx + SCGx (D′, D″) group. Scale bars: 100 μm. D: dentin; OB: odontoblast

**FIGURE A2:**
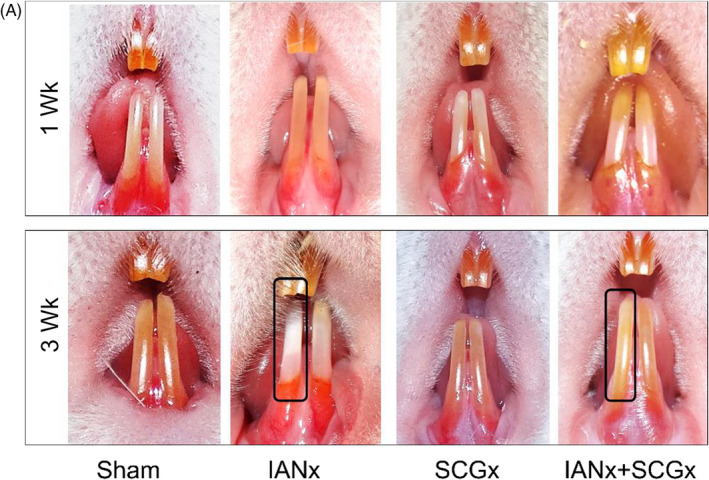
The incisor phenotype changes with time. A, After 1 wk of surgery, there were no differences in the four groups. After 3 wk of surgery, the right incisor of IANx and IANx + SCGx groups turn chalky (indicated by boxes)

**FIGURE A3:**
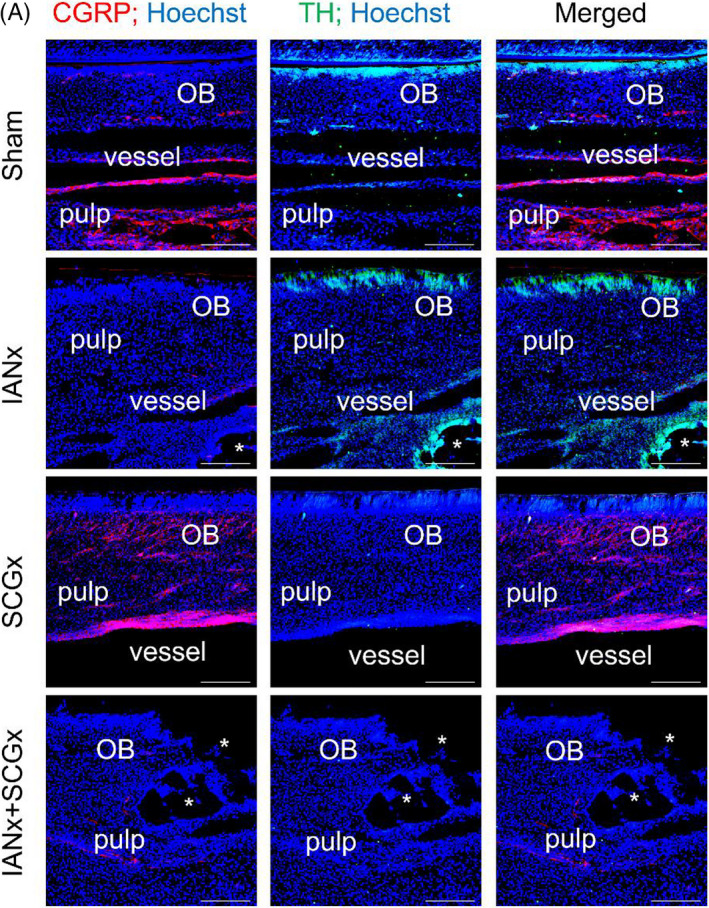
The efficiency of denervation. A, Immunofluorescence staining shows the expression of CGRP (red) decrease in IANx and IANx + SCGx group, while the expression of TH (green) decrease in SCGx and IANx + SCGx group. Scale bar: 20 μm, OB: odontoblast, * indicates disorganized structures

The rat lower incisors are mainly innervated by sensory nerve fibres derived from IAN and sympathetic nerve fibres derived from SCG. To clarify the role of sensory and sympathetic nerve in supporting tooth homeostasis, we established rat models including sectioning the unilateral sensory nerve (IANx) and sympathetic nerve (SCGx) (Figure A1). Firstly, we observed the incisors changes in 1, 2 and 3 weeks (Figure 1, Figure A2). Compared to Sham (Figure 1A) and SCGx group (Figure 1C), the incisors from IANx (Figure 1B) and IANx + SCGx group (Figure 1D) turned chalky after 2 weeks of surgery. Thus, we set 2 weeks after surgery as a time point to do further research. Micro‐CT of the four groups showed some irregular masses of calcifications in pulp of IANx (Figure 1F) and IANx + SCGx group (Figure 1H), but not in Sham (Figure 1E) and SCGx group (Figure 1G). To further confirm the effect of denervated surgery, we used calcitonin gene‐related peptide (CGRP) and tyrosine hydroxylase (TH) to label sensory nerve and sympathetic nerve respectively. The expression of CGRP decreased in IANx group, and the expression of TH decreased in SCGx group after two weeks of denervation (Figure A3). The immunofluorescence results confirmed the successful establishment of denervated incisor model. Additionally, we performed HE and Masson staining to observe the changes of incisors through longitudinal section. The IANx (Figure 2B(B″) and IANx + SCGx group (Figure 2D(D″) showed a disordered structure of pulp, dentin, enamel as well as periodontium compared to Sham (Figure 2A(A″) and SCGx group (Figure 2C(C″). The mixed structure was mainly composed of cells and irregular calcified masses which looked like true pulp stones or others called it osteodentin.^23^ The finding indicated that it is the sensory nerve, not sympathetic nerve, maintains the tooth homeostasis.

### 3.2 Sensory nerve contributes to dentin formation

**FIGURE 3:**
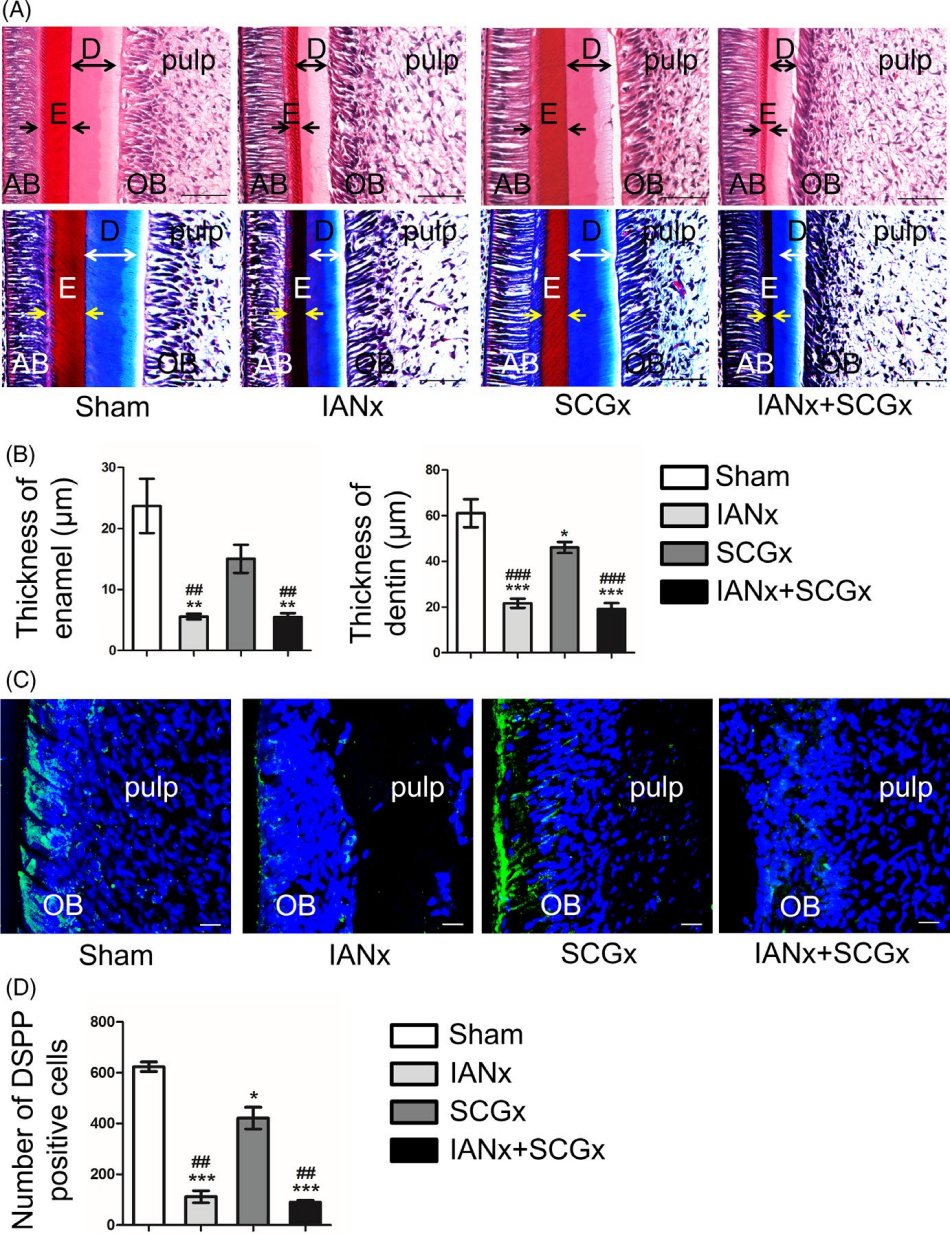
Sensory nerve contributes to dentin formation. A, HE and Masson staining shows the area of incisor apical at comparable positions. IANx and IANx + SCGx group exhibits thinner enamel (indicated by arrows) and dentin (indicated by double‐headed arrows) walls than Sham and SCGx group. Scale bars: 100 μm. B, The thickness of enamel and dentin in IANx and IANx + SCGx group dramatically decrease. (*P < .05, **P < .01, ***P < .005 vs Sham group. ##*P* < .01, ###*P* < .005 vs SCGx group, n = 6). C, Representative immunofluorescence images on the layer of odontoblast. Scale bars: 20 μm. D, The number of DSPP‐positive cells (green) decreased in the other three groups. (*P < .05, ***P < .005 vs Sham group, ##*P* < .01 vs SCGx group, n = 5). AB: adamantoblast; E: enamel; D: dentin; OB: odontoblast

Through micro‐CT analysis, we acquired cross‐sections through a fixed position to compare the thickness of dentin wall of the four groups. Compared to Sham group, the other three groups showed a reduction of dentin thickness (Figure 1I‐M, *P* < .05). Among them, the thickness of dentin wall in IANx and IANx + SCGx group displayed a significant decrease (Figure 1M, *P* < .01). Histological examination also exhibited distinct thinner layer of enamel and dentin, respectively, at the incisor apical of IANx and IANx + SCGx group (Figure 3A,B, *P* < .01), comparing with Sham and SCGx group. While comparing with Sham group, the SCGx group showed a slight decrease in dentin thickness as well (Figure 3A,B, *P* < .05). The staining of dentin sialophosphoprotein (DSPP), the specific marker of odontoblast, revealed lower number of DPSS‐positive odontoblasts in IANx and IANx + SCGx group than Sham (Figure 3C,D, *P* < .005) and SCGx group (Figure 3C,D, *P* < .01). SCGx group also showed a mild reduction of DPSS‐positive cells (Figure 3C,D, *P* < .05). The data above implicated that both sensory and sympathetic nerve paticipate in the process of dentin formation, but sensory nerve major governs the process.

### 3.3 Dental pulp stem cells from sensory nerve‐deficient microenvironment display impaired properties

**FIGURE 4:**
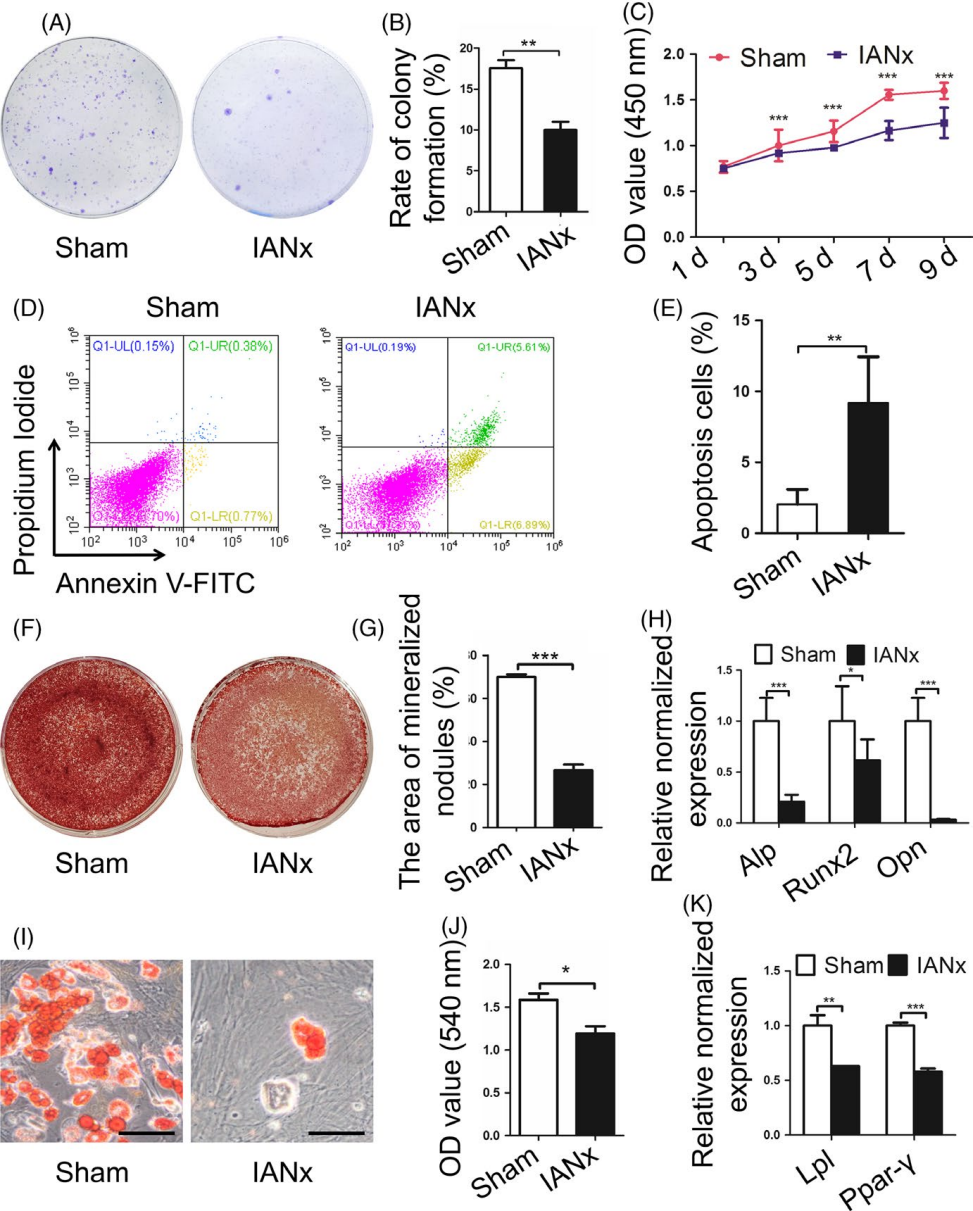
Dental pulp stem cells (DPSCs) from sensory nerve‐deficient microenvironment display impaired properties. A, B, The IANx group displays lower rate of colony formation than Sham group. (**P < .01, n = 4). C, The CCK‐8 assay shows the poor proliferative capacity of IANx group. (***P < .005, n = 5). D, E, The cell apoptosis assay exhibits higher rate of apoptotic cells in IANx group. (**P < .01, n = 4). F‐H, The IANx group displays impaired osteogenesis capacity in mineralized nodules formation (F, G) and osteogenic related genes *Alp, Runx2* and *Opn* (H). (*P < .05, ***P < .005, n = 6). I‐K, The IANx group shows impaired adipogenic potential in lipid droplets formation (I, J) and adipogenic‐related genes *Lpl* and *Ppar‐γ* (K). Scale bars: 50 μm. (*P < .05, **P < .01, ***P < .005, n = 6)

**FIGURE A4:**
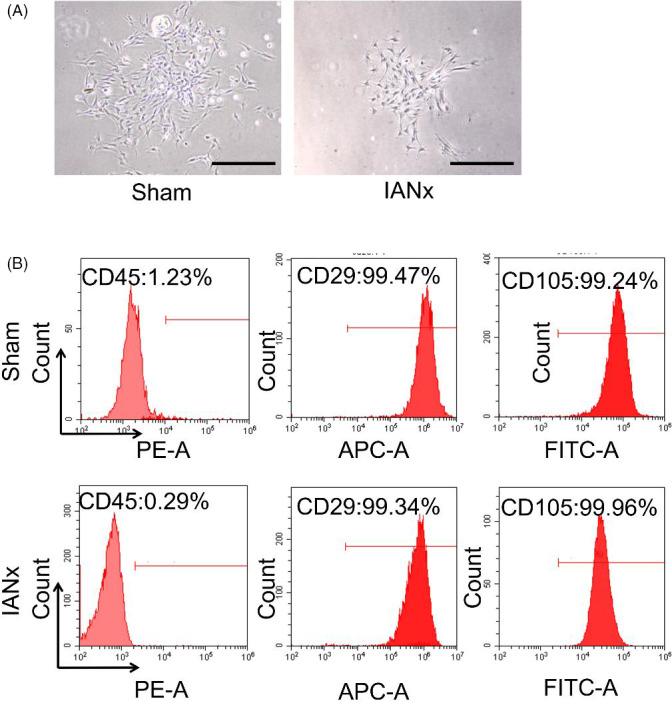
The morphology and characteristics of DPSCs. A, The Sham group exhibits a long spindle‐shaped morphology and grew robustly. However, the IANx group exhibits long spindle and polygonal shape without good growth state. Scale bars: 100 μm B, Flow cytometry analysis of DPSCs from IANx and Sham group shows that the MSCs surface markers CD29 and CD105 are positive of DPSCs, while the hematopoietic stem cell surface marker CD45 is negative

To investigate the influence of microenvironment lack of sensory innervation on DPSCs, we isolated the DPSCs from sensory nerve‐deficient microenvironment (IANx‐DPSCs). The DPSCs from Sham group (Sham‐DPSCs) were used as control. The Sham‐DPSCs exhibited a long spindle‐shaped morphology, while many IANx‐DPSCs exhibited polygonal shape (Figure A4(A)). Flow cytometry analysis of DPSCs from the two groups showed that they were both positive for MSCs markers CD29 and CD105 and negative for hemopoietic stem cells marker CD45 (Figure A4(B)). Moreover, the CFU analysis displayed that the IANx‐DPSCs exhibited lower CFUs rates than Sham‐DPSCs (Figure 4A,B, *P* < .01). In addition, compared to Sham group, IANx‐DPSCs showed an impaired proliferative capacity (Figure 4C, *P* < .005) and higher percentage of apoptotic cells (Figure 4D,E, *P* < .01).

To further compare the multi‐differentiation capacity of the two groups, we analysed the osteogenic and adipogenic differentiation capacities of DPSCs. The findings revealed that both groups possess osteogenic and adipogenic potential, while the IANx‐DPSCs displayed impaired osteogenesis and adipogenesis capacities. The IANx‐DPSCs showed less mineralized nodules (Figure 4F,G, *P* < .005) and lower expression level of osteogenic genes such as alkaline phosphatase (*Alp*) (Figure 4H, *P* < .005), runt‐related transcription factor‐2 (*Runx2*) (Figure 4H, *P* < .05) and osteopontin (*Opn*) (Figure 4H, *P* < .005) after osteogenic induction. Meanwhile, there were also less lipid droplets formation (Figure 4I,J, *P* < .05) and lower expression levels of adipogenic genes lipoprotein lipase (*Lpl*) (Figure 4K, *P* < .01) and peroxisome proliferator‐activated receptor‐γ (Ppar‐γ) (Figure 4K, *P* < .005) of the IANx group after adipogenic induction. The findings above showed sensory nerve‐deficient microenvironment leads to DPSCs dysfunction, implying the DPSCs retain the behaviour from the adverse microenvironment they derived from.

### 3.4 Supplementing activin B promotes proliferation and reduces apoptosis of DPSCs from denervated microenvironment

**FIGURE 5:**
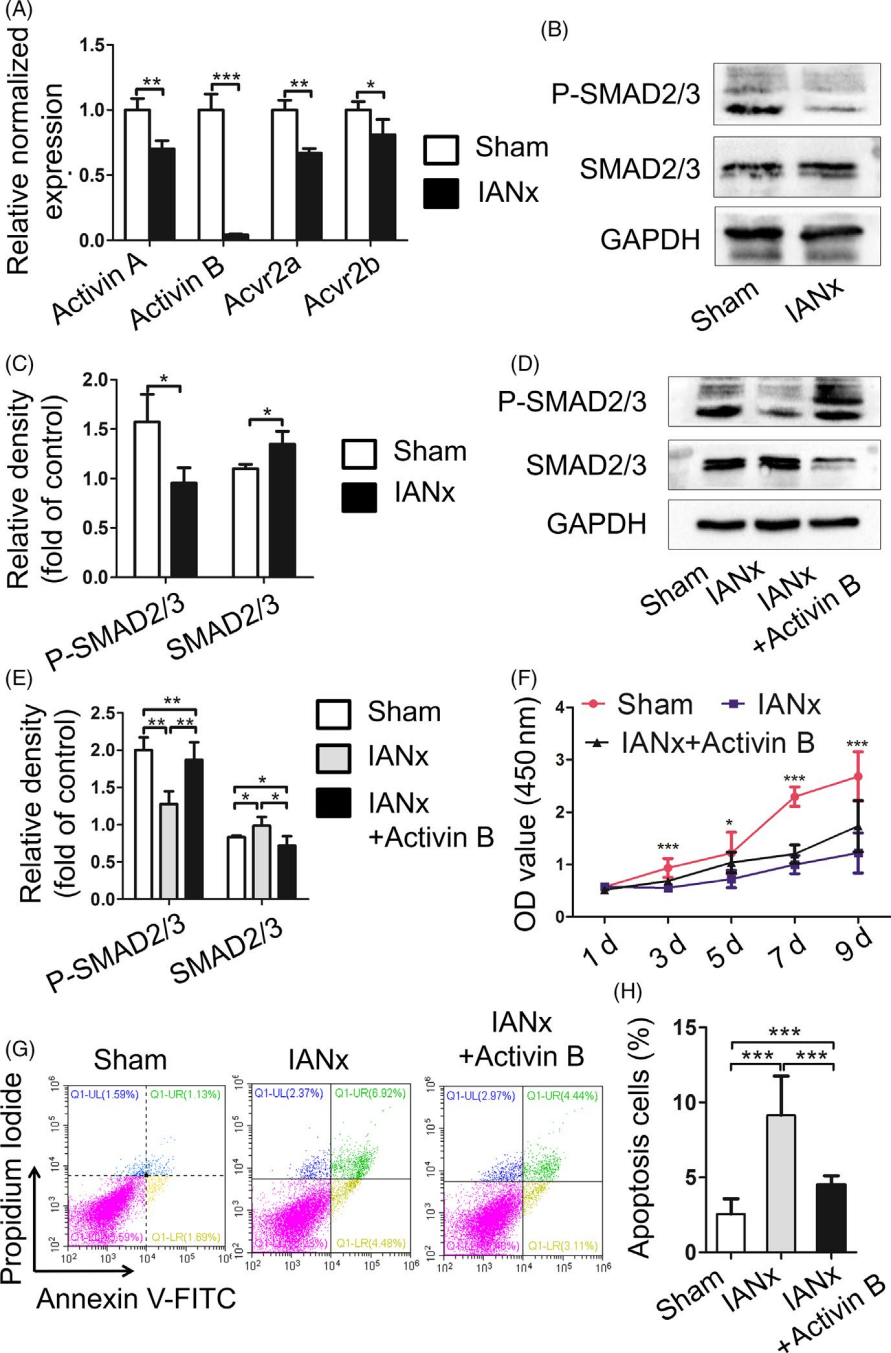
Supplementing activin B promotes proliferation and reduces apoptosis of DPSCs from denervated microenvironment. A, The expression level of *activin A, activin B, Acvr2a* and *Acvr2b* in pulp tissue from IANx and Sham group is measured by qRT‐PCR. All these gene expression levels from IANx group are lower than Sham group, especially *activin B*. (*P < .05, **P < .01, ***P < .005, n = 3). B, C, Western blot shows the expression level of phosphorylated SMAD2/3 decrease in DPSCs from IANx group. (*P < .05, n = 4). D, E, After activin B treatment, the protein expression level of phosphorylated SMAD2/3 increase in IANx group. (*P < .05, **P < .01, n = 4). F, CCK‐8 assay exhibits the proliferation increase in IANx group after supplementing activin B. (*P < .05, ***P < .005, n = 5). G, H, After activin B treatment, the IANx + activin B group displays lower rate of apoptotic cells than IANx‐DPSCs. (***P < .005, n = 4)

**FIGURE A5:**
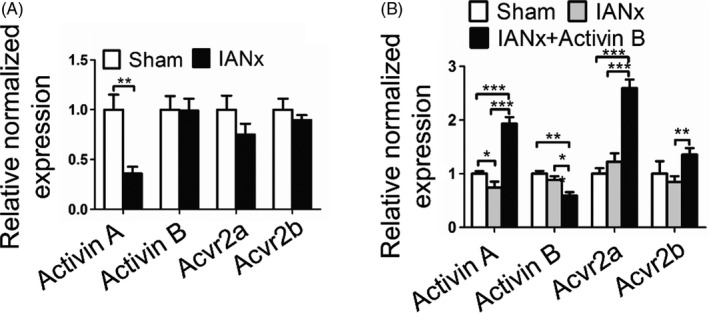
The activin signalling related genes in DPSCs. A, Except *activin A,* the expression level of *activin B, Acvr2a* and *Acvr2b* show no significant difference between IANx and Sham group. (**P < .01, n = 3). B, After activin B treatment, the expression level of activin A, Acvr2a and Acvr2b is increased in IANx group. (*P < .05, **P < .01, ***P < .005, n = 3)

Given sensory nerve‐deficient microenvironment impairs DPSCs properties, sensory nerve may secrete bioactive compounds to constitute favourable microenvironments for DPSCs. It has been shown that activin B as one of TGF‐β family ligands is expressed by sensory neurons and involved in microenvironments constitution.^24^ Therefore, we hypothesized that sensory nerve secretes activin B to modulate the biological behaviour of DPSCs. Firstly, we analysed the expression level of genes related to activin signalling (*activin A, activin B, Acvr2a* and *Acvr2b*) in pulp tissue from IANx and Sham group. Comparing with the Sham group, the expression level of *activin A, Acvr2a* and *Acvr2b* in IANx group exhibited a mild reduction (Figure 5A, *P* < .05), while the expression level of *activin B* had a dramatically decrease (Figure 5A, *P* < .005). Then, we evaluated the expression level of activin pathway‐related genes in IANx‐DPSCs and Sham‐DPSCs. The findings showed that despite the downregulation of *activin A* in IANx‐DPSCs (Figure A5(A), *P* < .01), the expression level of *activin B, Acvr2a* and *Acvr2b* had no statistical significance between the two groups (Figure A5(A), *P* > .05). Since the SMAD2/3 is the key transcription factors responding to activin B,^24^ we evaluated the expression level of SMAD2/3 and phosphorylated SMAD2/3 which import to nucleus driving the expression of downstream genes. Western blot analysis displayed that the level of SMAD2/3 was increased, whereas phosphorylated SMAD2/3 was reduced in IANx‐DPSCs (Figure 5B,C, *P* < .05), suggesting the deficiency of activin B in microenvironments results in long‐term downregulation of activin signalling.

To further confirm that activin B deficiency in microenvironments leads to DPSCs dysfunction, we applied activin B to IANx‐DPSCs culture medium after cell adhesion. The protein expression level of phosphorylated SMAD2/3 was increased in IANx + activin B group (Figure 5D,E, *P* < .01). The qRT‐PCR showed, except *activin B*, the levels of *activin A, Acvr2a* and *Acvr2b* were significantly elevated after activin B treatment (Figure A5(B), *P* < .05). Next, the CCK‐8 assay was used to analyse the proliferative ability among Sham, IANx and IANx + activin B groups. The data demonstrated treatment with activin B enhanced the proliferative capacity of IANx‐DPSCs since the third day of culture (Figure 5F, *P* < .005). Finally, the apoptotic cells were measured among the three groups. The IANx + activin B group showed a fewer apoptotic cells than IANx group (Figure 5G,H, *P* < .005). Taken together, these findings showed the impaired proliferative ability and higher apoptosis rate of denervated DPSCs could be ameliorated through replenishing activin B, illuminating sensory nerve secretes activin B to constitute favourable microenvironments for DPSCs, and supplementing activin B can partly rescue the impaired properties of DPSCs.

## 4 DISCUSSION

Nerve axons integrate in the tooth germ participating in tooth development.^25^, ^26^ However, how nerve regulates odontogenic stromal/stem cells to maintain tooth homeostasis remains elusive. The present study provides evidence for the first time that sensory nerve mainly contributes to tooth homeostasis, while sympathetic nerve only partly participates in the process of dentin formation. Furthermore, we first find that sensory nerve injury leads to disfunction of DPSCs via activin B/SMAD2/3 signalling in vitro. Supplementing activin B promotes proliferation and reduces apoptosis of DPSCs in sensory nerve‐deficient microenvironment.

Nerve is crucial in tooth development and homeostasis.^6^, ^26^ Our team previously has found abnormal morphology of teeth in a patient with congenital insensitivity to pain with anhidrosis (CIPA), which is a rare inherited disorder of the peripheral nervous system. The patient displays severe oral manifestations including dentin hypoplasia and cementogenesis defects,^26^ implying the indispensable role of sensory nerve in tooth development. For rodent lower incisor, it is innervated by sensory nerve fibres which come from IAN, sympathetic postganglionic fibres which origin from SCG and a few parasympathetic nerve fibres which are still under controversial.^25^ The sensory nerve primarily governs the sense of pain, the pressure and vasodilatation regulation,^4^, ^27^ while the sympathetic nerve tends to regulate vasoconstriction functions.^5^, ^28^ Though it has been reported IANx leads to slower incisor eruption and tooth discoloration,^23^ whether sympathetic nerve participate in incisor growth remains unclear. In this study, we utilized IANx, SCGx and IANx + SCGx models to investigate the contribution and interaction of sensory and sympathetic nerve in tooth growth. The results show the incisor from IANx + SCGx group displayed the similar changes with IANx group such as disorganized pulp structure, irregular calcification, as well as less dentin and enamel formation (Figures 1, 2, 3), indicating sympathetic nerve barely influences the tooth homeostasis. Although no obvious structure change was noted in the SCGx group, the thickness of dentin reduced comparing with Sham group, suggesting it participates in the process of dentin formation (Figures 1, 2, 3). To our knowledge, our study is the first to provide comparable evidence that it is sensory nerve, not sympathetic nerve, that mainly contributes to tooth homeostasis. The findings confirm the indispensable role of sensory nerve in tooth homeostasis and provide reliable evidence for further research.

In general, the inflammatory or neurodegenerative disease results in systemic changes, such as the capillary blood samples from patients with burning mouth syndrome show high level of oxidative stress.^29^ Microenvironment where the stromal/stem cells reside in is important in regulating cellular behaviour in multiple diseases and regenerative process.^30^, ^31^ The MSCs derived from diseased microenvironments such as inflammatory and oestrogen‐deficiency exhibit various degrees of impairments in stemness and regeneration capacity.^32^, ^33^ For example, the periodontal ligament stem cells (PDLSCs) from periodontitis microenvironment have a lower osteogenic, immunomodulatory and bone regeneration capability than those in healthy PDLSCs.^34^

It has been suggested nerve provides favourable microenvironment for stromal/stem cells.^35^, ^36^, ^37^ For instance, in bone marrow, sympathetic nerve is required for the construction of microenvironment and the mobilization of hematopoietic stem cells.^38^ Schwann cell precursors derived from peripheral nerve are reported to generate multipotent MSCs in tooth.^35^ However, whether the denervated microenvironment impedes DPSCs behaviour largely remains unknown. In this study, we isolated the DPSCs which is the MSCs in pulp to clarify the long‐term impact of denervated microenvironment on MSCs. Due to the sympathetic nerve barely influence tooth homeostasis, we only detected the difference between MSCs from Sham and IANx group. The data show although the DPSCs obtained from two groups both possess characteristics of MSCs (Figure A4), the DPSCs from IANx group exhibited obvious poor performance to maintain stemness (Figure 4). Given that stromal/stem or progenitor cells in pulp differentiate into odontoblasts and pulp cells to maintain tooth homeostasis, the impaired properties of DPSCs from sensory nerve‐deficient microenvironment may lead to disorganized tooth structure. The findings indicate the sensory nerve dependency of DPSCs and extend the understanding about the influence of microenvironment on MSCs, while further in vivo investigations are needed to confirm the phenomena.

The microenvironment where MSCs reside in modulates the biological behavior of MSCs.^10^ For example, 3D scaffolds promote cell survival and regenerative capacity.^39^ Application osthole promotes the impaired capacities of PDLSCs which come from periodontitis microenvironment.^21^ Supplementing the deficiency components of the denervated microenvironment provides a new pharmacological target for nerve dependent development and regeneration. However, how sensory nerve constructs microenvironment to support the MSCs in tooth remains largely unknown. In the present study, we find that although the adverse microenvironment has been removed, DPSCs from sensory nerve‐deficient microenvironment still display impaired capacities (Figure 4). Besides, the expression level of activin B dramatically reduced in IANx pulp tissue (Figure 5A), but not in DPSCs (Figure A5(A)). Importantly, supplementing activin B partly rescues the impaired properties of DPSCs (Figure 5F‐H), implying the low expression of activin B in denervated microenvironment impedes the behaviour of DPSCs.

The process of nerve navigation during tooth development is strictly regulated by signalling networks, such as semaphorin 3A,^40^ TGF‐β^41^ and activin signalling,^42^ and closely associated with tooth morphogenesis. Recent studies illuminate that in mice incisor, sensory nerve fibres secrete sonic hedgehog protein to activate Gli1^+^ cells, which is a critical subpopulation of MSCs differentiating into odontoblasts and pulp cells.^5^ Besides, activin signalling has been reported to be an early and essential mesenchyme signal for tooth development. activin proteins are formed by two subunit, activin βA and activin βB. The dimerization of the two subunits can produce activin A (βA: βA), activin B (βB: βB) and activin AB (βA: βB).^43^ Acvr2a and Acvr2b are two classical membrane receptors in activin signalling. Once activin proteins bind to receptors, intracellular SMAD2/3 could be induced and phosphorylated (activated). Then, phosphorylated SMAD2/3 will combine with SMAD4 and translocate to the nucleus regulating hundreds of genes transcription.^43^ Recently, a study has uncovered that activin B is specifically produced by sensory nervous system regulating microenvironment of hemocytes.^24^ However, whether activin B is important in the microenvironment of DPSCs has not been explored. In this study, the expression level of activin B dramatically decreases in IANx pulp tissue. Furthermore, DPSCs from denervated microenvironment show a downregulation of activin signalling which may be the reason for their poor performance (Figure 5A‐C). Application of activin B promotes the proliferation and decreases apoptotic cells of DPSCs in IANx + activin B group (Figure 5F‐H), indicating activin B may be secreted by sensory nerve to construct a favourable microenvironment for cells in it. The findings provide a new pharmacological target for related neurological disease. However, further experiments are required to determine whether activin B is involved in regulating stem cells behaviour and tooth homeostasis in vivo.

In summary, we first demonstrate that sensory nerve ablation has adverse impact not only on tooth homeostasis, but also on the properties of DPSCs. Though the DPSCs from sensory nerve‐deficient microenvironment exhibit impaired behaviour, they can be alleviated by activin B treatment. The present study exhibits that sensory nerve‐deficient microenvironment impairs tooth haemostasis by inducing apoptosis of DPSCs via activin B/SMAD2/3 signalling, providing a novel perspective for the interaction between sensory nerve and odontogenic MSCs.

## CONFLICT OF INTEREST

The authors have no conflicts of interest to declare.

## AUTHOR CONTRIBUTION

An‐Qi Liu, Li‐Shu Zhang and Dong‐Dong Fei contributed equally to the study design, manuscript preparation and data collection. Hao Guo and Mei‐Ling Wu made contributions to animal experiment. Jin Liu, Xiao‐Ning He and Yong‐Jie Zhang provided the analysis of in vitro study. Bei Li and Kun Xuan designed the experiments, oversaw the collection of results and data interpretation, and reviewed the final manuscript. All authors approved the final manuscript as submitted and agree to be accountable for all aspects of the work.

## Data Availability

The data sets used and/or analysed during the current study are available from the corresponding author on reasonable request.

## APPENDIX 1

### MATERIALS AND METHODS

#### Quantitative real‐time PCR (qRT‐PCR)

The relative gene expressions of osteogenic genes (*Alp, Runx2* and *Opn*), adipogenic genes (*Lpl* and *Ppar‐γ*) and activin signalling genes (*activin A, activin B, Acvr1, Acvr2a, Acvr2b*) in both pulp tissue and rDPSCs from Sham and IANx groups were tested by qRT‐PCR. The primers used are listed in below:

qRT‐PCR Primer Sequences

**Table:**

Genes	Primer sequence (5′–3′) (forward/reverse)	Product size (bp)
*Alp*	CTCCTTAGGGCCACCGCT	180
	GGTCCGATGGCCAGTACTAA	
*Runx2*	AACCAAGTGGCCAGGTTCAA	143
	GGACCGTCCACTGTCACTTT	
*Opn*	CCAGCCAAGGACCAACTACA	152
	CCAAGTGGCTACAGCATCTGA	
*Lpl*	CAGGATGTGGCCCGGTTTAT	189
	CGGGGCTTCTGCATACTCAA	
*Pparγ*	TCAGGTTTGGGCGAATGC	155
	GGCTTTGGTCAGCGGGAA	
*Actin A*	GTCACCATCCGTCTGTTTCA	231
	GCACTGTTCACAAGCAATCC	
*Actin B*	GCACCCACTGGCTACTACGG	156
	GGGGATGCAGCAAGAGTTCA	
*Acvr2a*	ACTTTTGTTGCTGTGAGGGC	283
	AGTAATGGGGAAGGTGGAGG	
*Acvr2b*	GGCATGAAGCACGAAAACCT	255
	GAAGTCCCTGTGGGCAATAGAA	
*GAPDH:*	CGAGACACGATGGTGAAGGT	166
	TGCCGTGGGTGGAATCATAC	

#### Western blot analysis

The total proteins from Sham, IANx and IANx + activin B groups were extracted with RIPA lysis buffer with protease inhibitor cocktail (Sigma) on the ice. The following primary antibodies were used in our study: Anti‐GAPDH Mouse Monoclonal Antibody (Cwbiotech, CW0100, 1:1000), P‐SMAD2/3 (Ser 423/425) Antibody (Santa Cruz Biotechnology, sc‐11769, 1:1000) and SMAD2/3 (D7G7) XP^®^ Rabbit mAb (Cell Signaling Technology, #8685, 1:1000).
